# Dairy as a Source of Iodine and Protein in the UK: Implications for Human Health Across the Life Course, and Future Policy and Research

**DOI:** 10.3389/fnut.2022.800559

**Published:** 2022-02-10

**Authors:** Oliver C. Witard, Sarah C. Bath, Mariana Dineva, Laury Sellem, Ana-Isabel Mulet-Cabero, Laura H. van Dongen, Ju-Sheng Zheng, Carina Valenzuela, Benoit Smeuninx

**Affiliations:** ^1^Centre for Human and Applied Physiological Sciences, Faculty of Life Sciences and Medicine, King's College London, London, United Kingdom; ^2^Department of Nutritional Sciences, Faculty of Health and Medical Sciences, University of Surrey, Guildford, United Kingdom; ^3^Hugh Sinclair Unit of Human Nutrition, Department of Food and Nutritional Science, University of Reading, Reading, United Kingdom; ^4^Food Innovation and Health, Quadram Institute Bioscience, Norwich Research Park, Norwich, United Kingdom; ^5^Division of Human Nutrition, Wageningen University and Research Centre, Wageningen, Netherlands; ^6^School of Life Sciences, Westlake University, Hangzhou, China; ^7^Faculty of Medicine, School of Human Development and Health, University of Southampton, Southampton, United Kingdom; ^8^Monash Institute of Pharmaceutical Sciences, Monash University, Parkville, VIC, Australia

**Keywords:** dairy, iodine, protein, pregnancy, sarcopenia

## Abstract

This narrative review summarizes key concepts in dairy nutrition for supporting human health throughout the life course. Milk and dairy products have been a staple component of our diet for thousands of years and provide a wide range of important nutrients that are otherwise difficult to obtain from dairy-free diets. In this review, we provide a broad perspective on the nutritional roles of iodine and dairy protein in supporting human health during pregnancy and early life, childhood and adolescence, mid- and later-life. New methodologies to identify biomarkers of dairy intake *via* high-throughput mass spectrometry are discussed, and new concepts such as the role of the food matrix in dairy nutrition are introduced. Finally, future policy and research related to the consumption of dairy and non-dairy alternatives for health are discussed with a view to improving nutritional status across the lifespan.

## Introduction

The clinical implications of dairy nutrition encompass somatic growth and development, neurological and cognitive development, and cardiometabolic health, including risk and incidence or cardiovascular disease (CVD), type 2 diabetes and sarcopenia. Hence, the nutritional value of dairy products is relevant across the lifespan. The over-arching aim of this narrative review is to summarize the state-of-the-art regarding the myriad of health implications associated with dairy intake across the life course, and how this science could be used to help shape future policy. The review is organized into three sections: (i) pregnancy, childhood and adolescence, (ii) mid life, and (iii) older age. While milk and dairy products provide a variety of nutrients that are important across the life course, for example calcium and vitamin B12, this review focuses on the health implications of the constituent protein and iodine content of milk and dairy products. We focus on these nutrients because they are of current concern from both the perspective of population deficiency risk, and because there are implications for these nutrients when plant-based milk-alternatives are used as a substitute to cows' milk, a practice that is becoming increasingly common in many countries, including the UK. For information on the health impact of other dairy-derived macro- and micronutrients, such as calcium, we refer the reader to other publications (1, 2). In this review, we also focus on new methodologies for assessment of dairy intake and considerations for future research into dairy nutrition and health, including vitamin D fortification.

## Dairy in Pregnancy, Childhood, and Adolescence

### Iodine

Milk and dairy products are the primary dietary source of iodine in the UK and many other European countries (3), especially where iodised salt is not mandatory or widely available. Iodine is essential for thyroid hormone production [tri-iodothyronine (T_3_) and thyroxine (T_4_)] which are crucial for brain and neurological development during pregnancy and early life. Iodine deficiency disorders include goiter (thyroid enlargement), impaired neurological development, and negative effects on child growth and development (4, 5). Iodine deficiency during pregnancy has considerable implications for the developing child and the iodine-intake recommendation is higher for pregnant/lactating women than adults according to WHO (250 vs. 150 μg/day for non-pregnant adults), although in the UK recommendation is the same (140 μg/day) (6, 7), as it is assumed that iodine stores are maximized prior to pregnancy, which can be used to meet additional demands (7). Emerging evidence suggests that even mild-to-moderate iodine deficiency during pregnancy may be negatively associated with child cognition and behavior (8, 9). This observation underpins the need for follow up interventional studies in pregnant women that utilize milk and dairy products as a vehicle to reverse iodine deficiency and improve the cognitive health of new-borns.

Cow's milk has a naturally low iodine concentration but is a rich source of iodine through standard farming practices such as the addition of iodine salts to cattle feed and use of iodine-based disinfectants (10–12). Iodophors mainly increase iodine concentration in milk through skin absorption of the iodine from topical application to teats (either pre or post milking) (13) and to a lesser extent through residues on the teat that transfer to the milk, especially if the teats are not dried sufficiently (14). In some countries, iodophors have been replaced with other disinfectants, and this is one explanation suggested for the decline in milk iodine concentrations in those countries [e.g. Australia and New Zealand (15, 16)]. Iodophors continue to be permitted and used in the UK, and in other countries. Indeed, a recent study in Switzerland found that teat dipping with iodine-containing disinfectants significantly increased milk-iodine concentration (17).

There is considerable variability in the iodine content of milk between countries, which is likely a result of differences in farming practice (12). UK milk has a relatively high iodine concentration compared with many other countries (12) at 427 μg/L (18). In the UK, a glass of milk (200 ml) provides 85 μg of iodine, constituting ~57 and 34% of the WHO recommended iodine intake for adults (150 μg/day) and pregnant women (250 μg/day), respectively (19). Seasonal variation in milk-iodine concentration exists whereby winter milk has a higher iodine concentration than summer milk (11, 20). This is a result of greater reliance on mineral-fortified feed during winter months when cattle are housed indoors rather than grazing on pasture (12), and possibly also related to goitrogenic components of feed being lower in winter milk (i.e., reducing competition with iodine and allowing greater excretion into milk). In the UK, and other European countries, studies have previously found also demonstrate that organic milk is lower in iodine content than conventional milk (~40% lower) (21, 22), largely as a result of restrictions on mineral fortified feed and higher goitrogen components of feed; however organic milk is still a good source of iodine in the UK, with a concentration of 241 μg/L and more recent UK research suggests that there is no overall difference in iodine concentration between organic and conventional milk (23), likely because of changes in organic farming methods. However, these sources of variation mean that milk is an inconsistent source of iodine, and the value assigned in food tables may be inaccurate, making it difficult to assess iodine intake and to provide dietary recommendations for optimal iodine intake.

Milk and dairy products have been shown to be determinants of iodine status in pregnant women. For example, in a study in three European countries [the Netherlands, Spain and the UK (24)], of all food groups, “milk and dairy products” was the only food group that was positively associated with iodine status. A UK study demonstrated that milk (out of a number of investigated dietary factors) was significantly associated with iodine status in pregnant women (25) (median iodine-to-creatinine ratio in women with milk intake 140 vs. 280 ml/day: 72 vs. 150 μg/g), whereas seafood intake was not (median iodine-to-creatinine ratio in women with seafood intake 2 vs. 2 portions/wk: 107 vs. 99 μg/g). The seasonal variability in milk-iodine concentration (18) translates to iodine status in pregnancy, with higher iodine status in winter months and the greatest difference between seasons in those who consumed the most milk (25). This observation underpins the need for a reliable and constant source of iodine so that individuals are not affected by underlying changes in milk-iodine concentration associated with farming practice.

Milk and dairy consumption contribute 51% of total iodine intake in UK children aged 4–10 years. A relatively high milk intake in children is one possible explanation for the iodine-sufficiency observed in UK children (4–10 years) in the National Diet and Nutrition Survey [NDNS (3)] and other UK studies (26). In contrast, there have been reports that 27% of girls aged 11–18 years have a low iodine intake (3) and pregnant women are classified as deficient (25). Given that milk intake, and thus iodine status, is higher in children than adults in countries where milk is an important iodine source is problematic for population monitoring. This issue stems from the WHO recommendation that population iodine status is monitored through assessment of school-aged children, however, this is likely to lead to an overestimation of population iodine status if a child's iodine intake (skewed by milk intake) is not representative of other population groups, such as women of childbearing age (27). Indeed, women of childbearing age are susceptible to low iodine intake and cross-sectional studies of adolescent girls (14–15 years) in the UK (*n* = 737) (28) and Ireland (*n* = 903) (29) demonstrate a positive association between milk intake and iodine status. In those studies, other dietary sources of iodine (such as fish) were not significantly associated with iodine status. Although fish (e.g., cod, haddock) is a rich natural source of iodine (30), in countries, such as the UK, these iodine sources make a relatively small contribution to population iodine intake [10 vs. 34% for milk and dairy products for adults 19–64 years (3)] because of their relatively infrequent consumption.

Historically, in the UK there was a concern that high consumers of milk would be at risk of excess iodine intake. This concern was especially true for young children, who have a higher milk intake than adults and who may exceed the Tolerable Upper Limit (TUL) for iodine from milk intake alone [TUL: 250 μg/d for 7–10 yr olds (31)]. Therefore, in the 1980s and 1990s, the milk-iodine concentration of UK milk was monitored by the (then) Ministry of Agriculture Fisheries and Food (MAFF), and estimated exposure was calculated for different age groups (32). Since then, the regulation for the permitted iodine content of cattle feed has been revised over concerns of potential toxicity of iodine from milk (33); the maximum concentration has been reduced from 40 mg/kg dry matter, to 5 mg/kg (33). The health implications of excess iodine intake in children are relatively unexplored but iodine excess can lead to both hyper- and hypo-thyroidism and should be avoided, especially in those with previous history of iodine deficiency (34).

### Protein

The consumption of adequate amounts of high-quality protein during childhood and early adolescence is crucial for optimizing growth and development (35). The role of milk consumption for growth and maturation in children is widely recognized in both developing and developed countries (36). For example, a trial in Bangladesh compared the growth of children in households which kept dairy cows with those that did not (37). The study reported that children in dairy cow owning households had increased height-for-age Z scores (+0.52 standard deviations) during the rapid growth period of 6- to 23-months of age compared with children in households without dairy cows. Moreover, children aged 0–11 months from the households with dairy cows were 21.7% less likely to be breastfed than those in households without cows. These findings suggest that easy access to cows' milk can substantially reduce the incentive for mothers to adopt breast feeding.

The benefit of milk consumption on linear growth during childhood is now understood to be primarily attributed to the casein fraction stimulating the secretion of hepatic insulin-like growth factor 1 (IGF-1) (38). Consistent with this notion, a recent study in pre-pubescent (8 years old) boys demonstrated that 7 days of casein administration increased serum IGF-1 concentrations to a greater extent than whey, with whey eliciting a greater insulin response (38). Moreover, the report of Locatelli et al. (39) highlights the mechanistic role of IGF-1 in the longitudinal growth of bone and skeletal maturation, as well as bone mass development and its remodeling in adult life. The regulation of bone length is linked to changes in chondrocytes of the proliferative and hypertrophic zones of the growth plate (40) and it now understood that activation of the Peroxisome Proliferator- Activated Receptor gamma (PPARγ) regulates changes in hepatic IGF-1 secretion and gene expression resulting from dietary protein intake (41). Allied with growth hormone (GH), IGF-1 mediates bone mass accretion by reducing collagen degradation and increasing the recruitment of osteoblastic cells and deposition of the collagen matrix (42). Furthermore, *in vitro* studies using chondrocytes have provided evidence that GH directly stimulates the formation of immature precondrocytes, whereas IGF-1 stimulates cells at a later stage of maturation. Hence, IGF-1 exerts a direct action on osteoblast function which is potentiated by the presence of GH and IGFBP-3 (insulin-like growth factor binding protein-3) (39). Taken together, these data provide important mechanistic insight regarding how dietary protein and related amino acids stimulate the hepatic secretion of IGF-1 and promote bone health during childhood.

Prepubertal males and females are in a state of constant growth, increasing stature by on average ~5 and 8.3 cm/y, respectively (43). Furthermore, the velocity of weight gain increases from 3 to 9 kg per year during puberty and almost entirely consists of lean mass. Underlying this growth in stature particularly during puberty is a chronic positive net muscle protein balance whereby rates of muscle protein synthesis (MPS) exceed muscle protein breakdown (MPB) (44). Traditionally, protein quality for muscle health is determined by the interplay of two factors, namely the digestive properties (and subsequent absorption kinetics) (45) and amino acid profile of an isolated intact protein source (i.e., whey, casein, soy protein). Collectively, these factors determine the bioavailability of protein-derived amino acids to the muscle for stimulation of MPS (46). Regarding amino acid composition, a complete profile of all indispensable (also known as “essential”) amino acids (IAA) is required to provide the building blocks necessary for MPS. Of the IAA, leucine serves as a key anabolic signal by activating the mechanistic target of rapamycin complex one pathway, leading to greater protein translational efficiency and ultimately increased MPS rates (47). Hence, it is generally accepted that rapidly digested protein sources that are rich in leucine content and contain a full complement of all IAA provide the highest quality protein. This has led to the general consensus that animal-derived protein sources are of higher quality than plant-derived protein sources. The potency of cow's milk to stimulate MPS in children aged 7–11 yrs has been shown by Karagounis et al. (48). In this study protein doses of ≥7 g at breakfast resulted in the attenuation of overnight protein losses in the subsequent 9 h. Furthermore, a protein dose-dependent increase in net muscle protein balance was observed, highlighting the importance of (milk-derived) protein intake with breakfast.

## Dairy in Mid-Life

### Iodine

Maintaining sufficient iodine levels during midlife is clinically relevant, especially for women of childbearing age who need to maximize thyroidal stores of iodine prior to pregnancy (49). Iodine stores can be used to maintain thyroid hormone production during periods of suboptimal iodine intake and increasing evidence suggests that long-term iodine sufficiency prior to pregnancy is preferable to an abrupt increase in iodine intake at the onset of pregnancy (50). Adults need to maintain thyroid hormone production through adequate iodine intake, not only to prevent thyroid enlargement (and eventually goiter) but also as iodine deficiency (especially moderate-to-severe) is associated with certain types of thyroid cancer (51) and hypercholesterolemia (52).

While data presented in the NDNS suggest that adults exhibit an adequate overall iodine status, a significant proportion of adult women have low iodine intake (i.e., intake below the Lower Reference Nutrient Intake) (53). In the UK, dairy consumption contributes 34% of total iodine intake in adults (19 to 64 years), and a positive association has been observed between milk intake and iodine status in women of reproductive age (54), underpinning the importance of milk as a source of iodine at this life stage. Dairy products provide an effective means to raise iodine status in women of childbearing age. Support for this notion is provided by a 12-week randomized controlled trial in women of childbearing age in Northern Ireland (55) that reported a higher iodine status in the intervention group (who were provided with additional milk) after 6 and 12 weeks compared with the control group (who continued their usual milk intake). Indeed, milk intake was 340 vs. 130 ml/day at 6 weeks and 260 vs. 120 ml/day at 12 weeks in the intervention and control group respectively) (55).

### Protein

The role of protein nutrition in maintaining skeletal muscle mass and strength is crucial for healthy aging (56). Numerous studies have demonstrated an acute stimulation of MPS with protein ingestion, with a graded protein dose-MPS response relationship observed up to 20–30 g of ingested high-quality protein (57). Most studies investigating the MPS response to protein ingestion have focussed on isolated protein sources such as whey, casein and soy. In these studies, the milk protein fractions (whey and casein) were shown to elicit a greater MPS response compared with plant derived proteins (58). Nevertheless, most individuals consume dietary protein in whole foods. In this regard, milk has been shown to promote muscle accretion to a greater extent than soy proteins when consumed after resistance exercise (59). Moreover, consuming an isonitrogenous dose of whole milk elicited a similar MPS response in middle-aged men when compared with whey protein, despite greater digestion rates and leucine availability with whey protein ingestion (60). A study by Burd et al. investigated the digestion and absorption kinetics, and subsequent MPS response, following the consumption of milk and beef in healthy young (18–35 y) men. Both protein sources increased the MPS response after resistance exercise, with milk eliciting a greater MPS response during the early postprandial phase (61).

A series of recent studies have investigated the response of MPS to the ingestion of alternative protein sources. For instance, a recent study demonstrated that ingesting a 35 g bolus of intact or hydrolysed wheat protein initiated a robust increase in circulating essential amino acid concentrations, however failed stimulate myofibrillar protein synthesis rates above basal values (62). Instead, a 60 g bolus of wheat protein was required to stimulate an increased response of MPS in older adults. Moreover, the ingestion of 30 g of corn protein (63) and 70 g of mycoprotein (64) was recently demonstrated to increase the stimulation of MPS in healthy young males. Taken together, these findings have implications for future dietary guidelines that might, besides supporting optimal nutritional guidelines, also support a more sustainable future for protein nutrition.

Whereas most studies have explored the amount of protein necessary to maximally stimulate MPS, a study by Mitchell et al. (60) investigated the minimal dose of milk protein concentrate required to enhance the anabolic signaling response to a bout of resistance exercise, concluding that 9 grams of milk protein was sufficient to enhance signaling proteins related to muscle protein anabolism. These studies highlight the capacity for dairy proteins, particularly milk and its respective protein fractions (whey and casein), to stimulate MPS to the same extent as isolated protein sources. Studies evaluating the efficacy of protein (dairy) nutrition to maintain skeletal muscle mass specifically during mid-life are scarce, but, to date, underscore their importance for healthy aging.

## Dairy in Later-Life (65 Years+)

### Iodine

Most studies that have investigated the impact of iodine deficiency on human health outcomes have been conducted in children and young adults; hence, data on the impact of iodine status in later life are currently limited. However, it has been hypothesized that long-term iodine deficiency may have a detrimental effect on cognitive function and brain volume in older age. In this regard, a study of 189 individuals in the Lothian Birth cohort demonstrated an association between low iodine intake (mainly related to low consumption of dairy products) and inner brain atrophy (65). However, there are inherent challenges with estimating iodine intake over many years, including fluctuations in milk-iodine content, meaning that these results are hypothesis-generating and require exploration in future research using a prospective-study design.

### Protein

A decline in skeletal muscle mass, strength and function (sarcopenia) is observed with advancing age and presents a clinical threat to independence by reducing mobility and increasing the risk of falls, fractures and hospitalization (66). Hence, the preservation of skeletal muscle mass is critically important for healthy aging. The causal mechanisms that underpin sarcopenia are clearly multifactorial, but ultimately stem from a chronic period of negative muscle protein balance (67). Contributing to this negative net muscle protein balance with age is the phenomenon termed “anabolic resistance” that describes the reduced capacity for older adults to mount a “youthful” MPS response to a meal-like (20–30 g) quantity of ingested protein (68). Hence, identifying high-quality protein sources that can stimulate a robust increase in MPS is crucial in mitigating sarcopenia and its associated morbidities.

Based on the superior quality of animal-derived protein sources, and in particular dairy, the postprandial MPS response to ingesting 20 or 40 g of whey protein (69) exceeded that of soy protein (70) in healthy older adults. Moreover, a recent study demonstrated a greater response of MPS to ingesting intact micellar casein compared with a dose-matched quantity of whole wheat; the most abundant plant-based protein in the diet comprising ~25% of total protein intake (62). The superior MPS response to the ingestion of milk proteins was primarily attributed to the more favorable IAA and leucine content of intact whey or casein compared with soy or wheat. Moreover, milk proteins are the only protein sources that exhibit a higher constituent IAA composition than human skeletal muscle, whilst boasting a complete IAA profile. In contrast, soy and wheat proteins are deficient in one or more IAA, namely methionine and lysine, and have lower leucine concentrations, rendering their IAA profile inferior to that of dairy proteins. Accordingly, based on typical dietary intake patterns across Europe (71) and North America (72), dairy holds a prominent position as a readily available and commonly consumed protein-rich food source for older adults in combating the threat of sarcopenia.

Specific protein intake guidelines for older individuals are currently lacking. The RDA of 0.8 g/kg/day for protein intake has recently been challenged and an alternative protein intake of 1.0–1.2 g/kg/day has been proposed (73). Furthermore, as a consequence of age-related anabolic resistance, the required per meal-protein dose is greater in older compared with younger individuals (74). To maximally stimulate MPS with each meal, older individuals require ~0.4 g/kg body mass of protein, whereas younger individuals require only 0.31 g/kg body mass of a high-quality protein (75). In the context of a mixed meal, a higher protein dose is likely necessary to maximally stimulate MPS. To aid the development of a framework for healthy aging, protein intake guidelines should be conceptualized not only in an age-specific but also in a meal-specific (breakfast, lunch, dinner and snacks) manner.

Whereas numerous studies have demonstrated a robust stimulation of MPS in response to ingesting an isolated intact protein source (i.e., whey, casein or soy protein), relatively few studies have compared the postprandial MPS response to different protein-rich foods. Moreover, limited information exists regarding how various food components modulate this process. This gap in knowledge has led to the recent emergence of evidence indicating a biological role for the food matrix in determining the anabolic capacity of commonly consumed protein-rich foods (76). As such, ingesting whole milk immediately following exercise resulted in greater muscle uptake of amino acids compared with an isonitrogenous dose of fat-free milk in young adults (77). These data suggest that an unidentified component or mechanism within the dairy matrix is able to increase the bioavailability of amino acids for MPS stimulation.

## Dairy Nutrition Across the Life Course–Future Research and Implications For Policy

### Research

Since there is considerable variation in the iodine content of milk in the UK, for instance because of season or organic farming (22, 23), further research is warranted to understand the factors that influence the iodine concentration of milk and how these may be manipulated to increase the reliability of milk as a source of iodine. For example, no data exist regarding the effect of key aspects of organic feed (such as white clover) on milk-iodine concentration, or whether the seasonal effects seen in the past are as pronounced today with the increasing practice for year-round housing for dairy cows on large farms. Furthermore, seaweed is starting to be used in farming (e.g., to reduce greenhouse gas emissions), either given to cows or used as a fertilizer on grassland; seaweed use in the dairy industry has been shown to increase milk-iodine concentration (78, 79) but further research on ways to incorporate it without risking iodine excess is required.

There also is a need for research to establish a reliable biomarker of individual iodine status so that risk of iodine deficiency can be established, both for future research studies and for clinical practice. Currently, the preponderance of evidence relies on urinary iodine concentration (which can be corrected for urinary creatinine concentration), but this is an imperfect marker on an individual basis and limits the exploration of associations between dietary intake and iodine status. Thus, establishing a more reliable biomarker, or combination of existing biomarkers (such as UIC with thyroglobulin measures) to provide a more robust assessment of long-term intake in individuals, would enable the impact of milk and dairy products on iodine status to be better understood, as well as identifying potential risk of deficiency in those who are not consuming iodine-rich foods.

With regards to protein, future research is warranted to elucidate the interaction between protein and other macronutrients on muscle metabolism. A study by Elliot et al. (77) showed a trend for increased uptake of the IAA phenylalanine and threonine when whole milk was provided after resistance exercise as opposed to fat-free milk and an isocaloric fat-free milk. These results are indicative of an interaction between the nitrogen utilization of the ingested protein and the other nutrients. The importance of the food matrix on MPS has been confirmed by van Vliet et al. that demonstrated a superior stimulation of MPS after resistance exercise when whole eggs, as opposed to egg whites only, were consumed (80). Therefore, future studies should investigate the effects of mixed nutrient meal ingestion on MPS rather than isolated protein sources.

### Policy–Milk Alternatives

Given that milk and dairy products are the primary source of iodine in many countries, including the UK, any policy that includes a shift away from milk and dairy products [e.g., based on the EAT Lancet report (81)] must consider adequate iodine intake and possibly alternative sources of iodine. Over the past 40 years, milk consumption in the UK has decreased, as demonstrated by data on the purchase of milk (82) and while the consumption of cow's milk exceeds other types of milk, the popularity of plant-based milk-alternative products (e.g., soya, almond, oat) has increased (83). For example, data from the US (84), Norway (85) and the UK (86) show that unless fortified with iodine, these milk-alternatives have a low iodine content compared with cow's milk (i.e., 7.3 vs. 438 μg/kg) (86). Most milk-alternative drinks on the UK market are not fortified with iodine (unlike calcium), although this may change in the future as more companies add iodine to their products.

The low iodine content of unfortified milk alternative products, coupled with the positive trend in consumption, might be particularly concerning in countries with limited availability of iodised salt, such as the UK. Indeed, in a study that extracted data from the UK NDNS (between 2014 and 2017; when most milk alternatives on the market were not iodine-fortified), individuals who exclusively consumed milk-alternative drinks had a significantly lower iodine intake (94 vs. 129 μg/day) and iodine status (measured by the median urinary iodine concentration (UIC): 79 vs. 132 μg/L) than cow's milk consumers, suggesting that consumers of milk alternatives were not replacing the iodine elsewhere in the diet (87). The results are meaningful from a public health perspective, as those who consumed cow's milk were classified as iodine-sufficient according to the WHO criterion (median UIC 100 μg/L), whereas the exclusive consumers of milk alternatives were classified as iodine deficient.

Changes in food patterns are likely to affect iodine status in the UK, particularly in specific population groups that are more likely to avoid and/or substitute cow's milk for milk alternatives (i.e., women of childbearing age, vegans, or individuals with a milk allergy) (83). Hence, it may be considered presumptuous to assume that milk alternatives are viable substitutes in the context of meeting dietary iodine recommendations across the life course, particularly in countries without an iodised salt policy. The nutritional content and micronutrient fortification of more recently emerging milk-alternative dairy products (e.g., cheese, yogurt, cream alternatives) and their impact on population nutritional status is unclear and should also be considered.

Manufacturers of plant-based alternative milk drinks (and other products sold as alternatives to dairy) should be encouraged to fortify their products with an appropriate amount of iodine. Indeed, the British Dietetic Association have recently launched a campaign to raise awareness about iodine and to ask for a commitment for iodine fortification from manufacturers of milk alternatives (88). The iodine content of these products should be similar to the (average) iodine concentration of milk so that consumers are not put at risk of iodine deficiency. Fortification should be with potassium iodide/iodate, rather than a seaweed-based ingredient in order to ensure reliable iodine concentration and avoid risk of excessive iodine.

In recent years, advocates of commonly consumed animal-based protein sources, including dairy, have been challenged by a social movement attributing, in part, the increase in worldwide greenhouse gas emissions (GHGE) to livestock production (89). Consistent with this idea, on a per gram basis, plant-derived proteins are associated with lower GHGE (<4 kg of carbon dioxide per kg of edible weight) than most animal-derived protein sources, including dairy (90). However, as outlined above, the reduced potency of plant-based proteins to stimulate MPS means that, in theory, a considerably larger amount of plant-based protein food is required to support the maintenance of muscle mass with aging. Furthermore, most plant proteins are high in fiber which impairs the digestive properties of the protein source, and are deficient in at least one IAA (91). Accordingly, if GHGE are expressed relative to the amount of food required to satisfy daily IAA requirements, the benefits of a plant-based protein diet over an animal-derived protein diet, including dairy, may become less clear-cut than is often espoused (92).

If milk and dairy products are not consumed (for various reasons), public-health messages need to ensure alternative sources of key nutrients are clearly signposted. In the case of iodine, other dietary sources would include fish and eggs, or appropriately fortified milk-alternative products. Although seaweed is a rich source of iodine, intake of brown seaweeds and supplements (e.g., kelp) can lead to excess iodine intake and therefore should be avoided as an iodine source (93).

### Potential Fortification of Milk and Milk Alternatives With Vitamin D

Milk and dairy products are not rich sources of Vitamin D. In this regard, UK cows' milk has trace concentrations (94) or 0.06 μg/100 ml according to recent analysis of whole milk in Northern Ireland (95). In contrast to iodine, milk-alternative drinks are often fortified with vitamin D, at approximately 0.75 μg/100 ml, which means that they are a better source of Vitamin D than cows' milk in the UK. In the US and Canada milk is fortified with vitamin D to a concentration of 0.875–1.125 μg/100 ml) (96). A recent study that modeled (using NDNS data) the impact of vitamin D fortification of milk at varying concentrations (1 μg/100 ml, 1.5 μg/100 ml or 2 μg/100 ml) showed that fortification had the greatest effect in children (1.5–3 years) with predicted vitamin D intake increasing to 4.8, 6.2, and 7.6 μg/day, respectively (95) [the vitamin D recommendation for is 10 μg/day (400 IU) (97)]. These data suggest that vitamin D fortification of milk could be a strategy to address vitamin D deficiency. This is significant as Vitamin D is essential for bone formation through its role in intestinal calcium absorption (98) and low vitamin D status can lead to rickets in children and a subsequent increased risk of osteoporosis in later life. At the beginning of the 20th century, around 80% of children in North America and Europe had severe rickets but was essentially eradicated by the late 1930s due to food fortification with vitamin D and provision of cod liver oil to children (99). Today, a sub-optimal vitamin D status is widespread in children and adults and it is of concern that cases of rickets are again increasing in the US (100), UK (101) and worldwide (102, 103) and vitamin D deficiency is similar for older adults as children, teenagers and middle-aged adults (104).

Maintaining adequate vitamin D levels (>50 nmol/L) in older individuals is important for bone health, muscle maintenance and several other health-related outcomes. Low plasma levels of 25(OH)D are associated with a higher risk of osteoporosis and is a key underlying factor for fractures in women and men aged >50 yrs (105). A study by Sahni et al. examined the association between dairy consumption and bone loss in older individuals and examined whether these associations were modified by vitamin D supplementation (106). The findings revealed a protective effect of dairy consumption on bone mineral density in those individuals who supplemented their diet with vitamin D. Taken together, these findings highlight the potential role of fortified dairy-derived vitamin D on bone health.

Recent evidence also suggests a potential role for vitamin D in the maintenance of muscle mass with advancing age (107, 108). A meta-analysis investigating the impact of dairy-derived protein and vitamin D supplementation demonstrated a positive effect of protein on body weight, whereas vitamin D facilitated small, but physiologically relevant improvements in physical performance in frail, inactive older individuals (109). Whilst acknowledging the value of fortifying dairy products with vitamin D, this intervention might not be adequate to fulfill daily vitamin D needs. Therefore, additional food sources, such as eggs (110) and orange juice (111) are often fortified in vitamin D and could contribute to overall adequate vitamin D status.

## New Concepts and Methodologies in Dairy Research

The accurate measurement of dairy intake represents a distinct challenge for epidemiological studies investigating the association between dairy intake and cardio-metabolic health across the life course. In this regard, identifying biomarkers of dairy intake is generally accepted to be an important strategy to overcome the limitations of traditional dietary assessment methods.

### Biomarkers of Dairy Fat

Several potential biomarkers of dairy fat have been explored including individual fatty acids such as odd-chain saturated pentadecanoic (C15:0) and heptadecanoic (C17:0) acids, along with ruminant trans-fatty acids such as trans-palmitoleic acid (t16:1n-7). Limited studies have assessed the relationship between reported dairy intake and C15:0 or C17:0 circulating and adipose tissue levels, and consistently favor C15:0 as the better biomarker of dairy fat compared to C17:0 (112). Moreover, circulating trans-palmitoleic acid was strongly correlated with whole-fat dairy products in a large American prospective study (113). However, these circulating fatty acids may respond to non-dairy dietary sources (e.g., fish, meat), endogenous synthesis and other nutrient intakes such as dietary fiber (114). Thus, the reliability of these results in the context of varied study populations and dietary patterns remains unclear.

### Biomarkers of Dairy Proteins

The limited number of studies that have explored potential biomarkers for dairy protein have produced mixed results. In a randomized controlled cross-over trial (115), 47 participants consumed meat-derived protein, dairy products or grain. Investigators identified potential urinary and plasma biomarkers for meat- and grain-derived protein but not for dairy protein. Potential biomarkers of whey protein (a globular protein isolated from whey) and casein (a major component of cheese) protein intake were identified in several other randomized cross-over studies, including linear dipeptides and γ-glutamyl conjugates (116). Nevertheless, these trials were small-scale, and more validation studies are warranted to confirm these results. In addition to dairy fat and protein biomarkers, other potential dairy biomarkers relate to gut microbiota, which could potentially provide signatures of specific dairy product intake. However, a paucity of studies has been conducted to evaluate the gut microbiota as a “biomarker” of dairy intake, and more prospective cohort studies or randomized trials are warranted.

### Untargeted Approaches to Identify Novel Biomarkers: Metabolomics and Lipidomics

Odd-chain fatty acids and trans-16:1n-7 have been consistently associated with dairy consumption and offer promising biomarkers of dairy fat intake. However, their limitations suggest a need for identifying novel biomarkers. Contrary to the candidate biomarker approach, the exponential increase in the use of metabolomics and lipidomics over the last few decades due to the development of high-throughput methods including proton nuclear magnetic resonance (H-NMR) and mass spectrometry (MS) offers a promising hypothesis-free approach to identify novel nutritional biomarkers (117). Metabolomics is the study of low-molecular weight metabolites (usually <1,500 Da) (117). Several studies have assessed the metabolomic profiles of blood (118–121), urine (122–126) or feces (125) samples, using MS (118–120, 122, 123) or H-NMR (121, 123–126), in a trial (119–126) or observational (118) study design among healthy adults, patients (120, 121) or children (124). More recently, Drouin-Chartier et al. (127) investigated numerous (*n* = 385) plasma metabolites in relation to dairy consumption among participants from the PREDIMED intervention study (*n* = 1,833) and a confirmatory cohort of 4,932 participants from the PREDIMED study year 1, the Nurses' Health Study, the Nurses' Health Study II, and the Health Professionals Follow-Up Study. This study identified 38 metabolites associated with total dairy consumption declared in food frequency questionnaires [Pearson correlation, r (95%CI) = 0.37 (0.33–0.40)] and 30 metabolites associated with intakes of reduced fat dairy [r = 0.24 (0.19–0.30)]. Among the metabolites measured, higher plasma levels of C14:0 sphingomyelin and C34:0 phosphatidylethanolamine, together with reduced levels of γ-butyrobetaine, were consistently associated with higher dairy intake. Furthermore, authors observed an inverse association between a score based on identified dairy-related metabolites and incident risk of type 2 diabetes in the PREDIMED baseline cohort [HR per 1 SD increment of metabolite score = 0.76 (0.63–0.90)], but not among the follow-up PREDIMED cohort or the US cohorts investigated, providing new evidence on potential biomarkers to study the mechanisms underlying the relationship between dairy nutrition and type 2 diabetes.

Whilst these studies have successfully utilized untargeted approaches to identify metabolite signals for dairy consumption, several limitations warrant consideration. As such, the inclusion of patient populations (120, 121) limits the generalisability and the identification of unspecific biomarkers of dairy consumption (122–126). The use of metabolomics for dairy consumption biomarker discovery is promising but still in its infancy, and the identified metabolites thus far still have their limitations. Therefore, further metabolomics studies are needed using a combination of metabolites in order to contribute toward the discovery of novel and more specific biomarkers of dairy consumption.

### Food Matrix Effect

The health and nutritional properties of foods are conventionally assessed according to their individual nutrient composition. This approach associates one nutrient with one health effect, e.g., the diet-heart hypothesis. According to this hypothesis, saturated fat foods, such as dairy, are often associated with higher cholesterol levels. However, a growing body of evidence suggests that postprandial responses are strongly affected by the food matrix (128), which is the arrangement of the food constituents and their interactions at the multiple length scales (129), e.g., from the molecular to the physical structure. This matrix is created naturally or modified by processing and can affect nutrient bioavailability and, subsequently, the metabolic responses.

Dairy products often provide the predominant dietary source of saturated fat. Historically, dietary fat intake has been linked with an increased risk of cardiovascular disease (CVD) resulting from elevations in serum concentrations of cholesterol and low density lipoprotein cholesterol (LDL-C) (130). This association has led to many dietary guidelines proposing a restriction of saturated fat intake to <10% of total energy intake (131). Despite being a major source of saturated fatty acids, there is now a substantial body of evidence from prospective studies and associated meta-analyses that suggest the overall intake of dairy foods is not associated with an increased risk of CVD. Indeed, a recent study reported a reduced risk of stroke and type 2 diabetes (132). However, there is an increasing body of evidence that supports the idea that specific fatty acids, rather than total saturated fatty acids, may exacerbate the adverse effects on CVD risk (133).

A key feature of any food source is the possible influence of the so-called food matrix that can modulate the amount of fat digested and absorbed. This notion was elegantly demonstrated by Hjerpsted et al. (134). In this study, 23 participants underwent two 6-week crossover periods whereby a proportion of their habitual dietary fat intake was replaced by either cheese or butter, both of which provided 80 and 36 g/day of total fat and saturated fatty acids, respectively. The calcium content of cheese and butter was 834 and 19 mg/100 g, respectively. Relative to baseline, cheese intake was not associated with an increase in serum total cholesterol or LDL-C concentrations, whereas the butter diet was associated with an increase in both lipids. Cholesterol and LDL-C concentrations in the cheese diet were 5.7 and 6.9% respectively lower than the butter diet. Further evidence for the food matrix effect can be found in a review by Thorning et al. (128).

Several mechanisms have been proposed to underpin the health benefits of manipulating food matrix. For instance, studies that provided a hard cheese resulted in an increased fecal fat and calcium excretion. This observation stemmed, at least in part, from the saponification reaction in the gastro-intestinal tract between calcium and fatty acids that resulted in the production of largely indigestible soaps. In addition, several studies have demonstrated increased fecal bile acid excretion that may be linked with their absorption onto amorphous calcium phosphate. An increased fecal bile acid excretion is indicative of reduced enterohepatic recycling of bile acids. Hence the liver synthesizes bile acids from cholesterol, and reduces bile acid recycling that may lead to reduction in circulating cholesterol concentrations (128). Finally, there is evidence that in some dairy foods the milk fat globule membrane can protect the dairy fat from digestion and absorption (135).

## Conclusions

Milk and dairy products provide important nutrients across the life course, some of which (e.g., iodine) are likely underappreciated by the consumer. Milk and dairy products (as a rich source of dietary iodine) intake should remain a staple component of the diet for pregnant/lactating women, given that iodine deficiency during pregnancy is associated with cognitive implications for the developing child. However, excessive iodine intake may be of concern in those who consume high quantities of iodine-rich milk (e.g., winter UK milk), and hence it is important to continue to monitor milk-iodine concentration, especially in relation to changes in the iodine content of cattle feed. Milk proteins, and specifically the casein fraction, are fundamental to supporting musculoskeletal growth and development during childhood and adolescents, mediated primarily by the secretion of IGF-1. With advancing age (mid and later life), dairy proteins play a prominent role in maintaining skeletal muscle mass and strength *via* the stimulation of MPS. Likewise, maintaining a sufficient iodine status with advancing age is particularly relevant given the emerging link between iodine deficiency and brain health in older age. Moving forward, it is critically important that policy and practice reflects the role that milk and dairy products play in the provision of important nutrients for metabolic health in humans across the life course.

## Author Contributions

All authors were involved in conceptualization, writing and editing of the narrative review and have read and agreed to the published version of the manuscript.

## Conflict of Interest

The authors declare that the research was conducted in the absence of any commercial or financial relationships that could be construed as a potential conflict of interest.

## Publisher's Note

All claims expressed in this article are solely those of the authors and do not necessarily represent those of their affiliated organizations, or those of the publisher, the editors and the reviewers. Any product that may be evaluated in this article, or claim that may be made by its manufacturer, is not guaranteed or endorsed by the publisher.
